# Characteristics of the shark fisheries of Fiji

**DOI:** 10.1038/srep17556

**Published:** 2015-12-02

**Authors:** Kerstin B. J. Glaus, Irene Adrian-Kalchhauser, Patricia Burkhardt-Holm, William T. White, Juerg M. Brunnschweiler

**Affiliations:** 1 Research Group Man-Society-Environment, Department of Environmental Science, University of Basel, Vesalgasse 1, 4051 Basel, Switzerland; 2 Department of Biological Sciences, University of Alberta, Edmonton, Canada; 3 CSIRO Australian National Fish Collection, National Research Collections Australia, GPO Box 1538, 7001 Hobart, Australia; 4 Independent Researcher, Gladbachstrasse 60, 8044 Zurich, Switzerland

## Abstract

Limited information is available on artisanal and subsistence shark fisheries across the Pacific. The aim of this study was to investigate Fiji’s inshore fisheries which catch sharks. In January and February 2013, 253 semi-directive interviews were conducted in 117 villages and at local harbours on Viti Levu, Vanua Levu, Taveuni, Ovalau and a number of islands of the Mamanuca and Yasawa archipelagos. Of the 253 interviewees, 81.4% reported to presently catch sharks, and 17.4% declared that they did not presently catch any sharks. Of the 206 fishers that reported to catch sharks, 18.4% targeted sharks and 81.6% caught sharks as bycatch. When targeted, primary use of sharks was for consumption or for sale. Sharks caught as bycatch were frequently released (69.6%), consumed (64.9%) or shared amongst the community (26.8%). Fishers’ identification based on an identification poster and DNA barcoding revealed that at least 12 species of elasmobranchs, 11 shark and one ray species (*Rhynchobatus australiae*) were caught. This study, which is the first focused exploration of the shark catch in Fiji’s inshore fisheries, suggests that the country’s artisanal shark fisheries are small but have the potential to develop into larger and possibly more targeted fisheries.

Compared to industrial fisheries, there has been a paucity of research on small-scale and artisanal fisheries until the past decade^1^. Artisanal fisheries provide an important source of food and employment, particularly in low-income countries^2^,^3^,^4^,^5^. In stark contrast to industrial fisheries, they are small-scale, typically subsistence in nature, and fishing effort is often unmonitored by regional fishery authorities due to a shortage of trained personnel and/or equipment (i.e. patrol boats) or more generally, a lack of resources^6^,^7^,^8^. As a result, the catch composition and biological characteristics of artisanal landings are often unknown and data on the extent and fate of bycatch is lacking^9^.

Sharks are an important component of marine ecosystems and are becoming increasingly threatened by overfishing^10^. Fishing pressure on sharks comes from both industrial and artisanal fisheries, which either target sharks or catch them, sometimes in substantial quantities, as bycatch. Several studies have reported that threatened elasmobranch species are caught as bycatch in artisanal fisheries^11^,^12^,^13^. Bycatch retained is often also a valuable source of income and, in some instances, can be a highly desirable component of the catch^14^. Whilst research efforts tend to focus on quantifying shark catches, catch composition and bycatch rates in industrial fisheries^15^,^16^,^17^, similar data are typically lacking for artisanal fisheries^18^. However, in developing countries, these small-scale artisanal fisheries can account for a substantial proportion of the overall fisheries capture production^19^.

Limited data on artisanal shark fisheries are available, at least for certain areas and specific fisheries, for the Saudi Arabian Red Sea^20^, the United Arab Emirates^21^, Mexico^22^,^23^,^24^, Indonesia^11^,^25^, Madagascar^26^,^27^,^28^ and Oman^29^,^30^. These studies highlight the importance of sharks and their products to these artisanal fisheries as well as the general lack of accurate catch and size composition data from these fisheries. Evidence from other parts of the world suggests that artisanal fishers profit from the sale of shark fins^31^,^32^,^33^. Since artisanal fishers comprise more than 95% of the world’s fishers^34^, there is an urgent need for baseline data on the extent of shark fishing on a global scale, both as target and as bycatch, in artisanal fisheries. If such data are not available, population declines are likely to go undetected and local authorities will have inadequate information for drafting management plans and implementing them in a timely manner.

Accurate species identification is one of the most significant issues needing to be addressed when assessing artisanal fisheries, particularly when only parts of the animals, e.g. shark fins, are retained. In these instances, molecular analyses have proven to be a powerful tool to assist identification of elasmobranch species by fishers^35^,^36^. One of the most widely used gene markers for species identification is cytochrome c oxidase 1 (CO1)^36^. In a study on 210 Australasian chondrichthyans, the CO1 gene permitted discrimination of 99% of these species^36^.

In the Pacific, subsistence and artisanal fishing including for sharks date back thousands of years^37^, and are vital to food security^38^. Throughout Oceania, many subsistence and small-scale commercial fisheries for sharks exist, but catches are generally poorly documented^39^. Fiji is no exception. The country’s subsistence and artisanal inshore coral reef fisheries target a wide range of vertebrate and invertebrate species typically using hand line, spear, gillnet, seine net, hookah (diving with surface supplied air), and reef gleaning^40^,^41^. Coastal fisheries production provides extensive benefits to Fijian communities, including employment and nutrition^40^. Sharks are fished both within the artisanal sector and taken as bycatch in the industrial tuna fishery^42^,^43^. While it is known that sharks are taken in offshore waters and the country exports shark fins to the international market^44^, there is limited information on how many sharks and of what species are caught in Fiji’s inshore reef fisheries^43^,^45^. This initial study aimed to provide baseline information on Fiji’s artisanal fisheries which catch sharks and rays. In addition to interviews conducted with fishers, we also used molecular species identification tools to determine and verify what shark species are caught, both as target and as bycatch.

## Methods

### Study Area and Interviews

**Figure 1:**
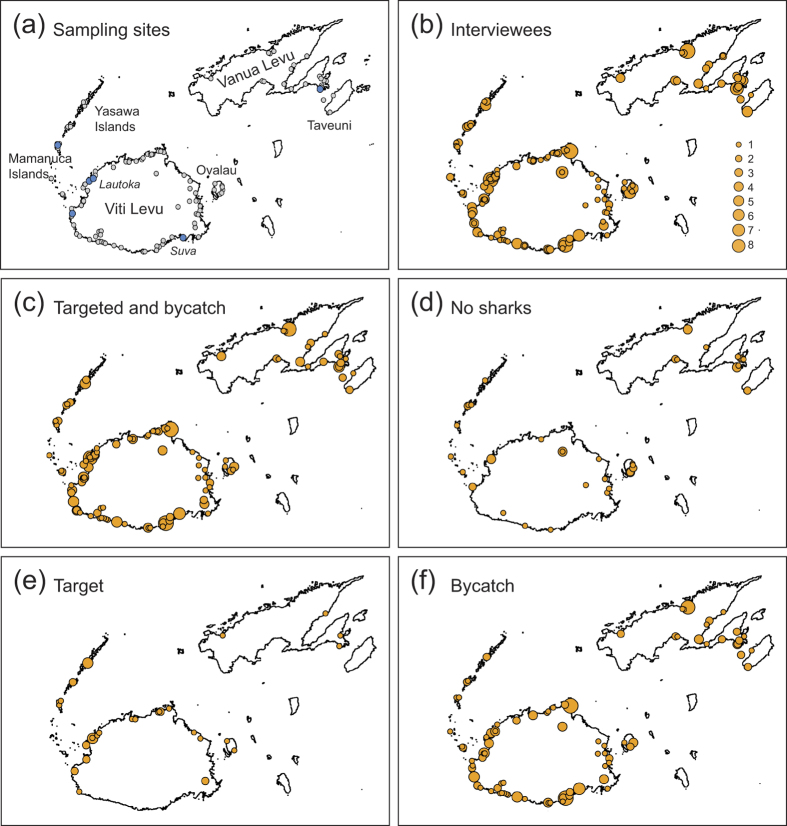
Interview sites and quantification of artisanal shark fisheries in Fiji. (**a**) Sites where interviews were conducted (n = 117; grey dots) and samples taken for DNA barcoding (n = 6; blue dots); bubble plots of (**b**) the number of interviewees, (**c**) the number of interviewees that reported to catch sharks (targeted and bycatch), (**d**) the number of interviewees that reported to never catch sharks, (**e**) the number of interviewees that reported to target sharks, and (**f**) the number of interviewees that reported to catch sharks only as bycatch. The area of each dot (**b–f**) corresponds to the number of interviewees at this site (see (**b**) for scale of bubble sizes). Maps were generated using the map function of R on publicly available coastline coordinates obtained from the NOAA National Geophysical Data Center (http://www.ngdc.noaa.gov/mgg/shorelines/shorelines.html).

The study area included the majority of the coastline of the main islands of Viti Levu and Vanua Levu, and Taveuni, Ovalau and islands of the Mamanuca and Yasawa archipelagos (Fig. 1a). Information was gathered by means of semi-directive interviews^46^,^47^ with fishers in 117 villages and at local harbours in Suva and Lautoka in January and February 2013. Suva is the capital and Lautoka the second largest city of Fiji. Lautoka is in the west and Suva on the southeast coast of the island of Viti Levu, Fiji’s largest island (Fig. 1a). Both cities have regular fish markets with multiple stands located close to the respective harbour where fish are landed. Since fishers were the only target group, an opportunistic sampling procedure was applied. Opportunistic sampling is a type of non-probability sampling where interviewees are selected based on naturally occurring groups^48^.

One of the authors (K.G.) visited all 117 villages and local harbours in Suva and Lautoka. She was accompanied by a male for interviews on Viti Levu, a female for village visits on Vanua Levu, Taveuni and Ovalau, and another female individual for interviews with fishers on the Mamanuca and Yasawa archipelagos. All three local Fijian collaborators were both fluent in English and Fijian (Bauan dialect). English is an official language in Fiji and interviews were conducted in English and Bauan, a common dialect which is used throughout Fiji. As per village protocol, permission was requested from village chiefs to interview fishers in their respective village. Chiefs would then designate fishers that could be interviewed. If no fishers were in the village at the time of our visit we were allowed to interview their wives who are fishing on a regular basis themselves and/or accompany their husbands. Like in many Pacific Island countries, in Fiji, women are the major contributors of subsistence food production^49^. The major fishing grounds for women are typically areas closer to shore compared to men who fish mostly from boats in deeper waters and do underwater diving^50^. Five female interviewees were interviewed by K.G. together with the male Fijian collaborator. All other interviews with females (n = 6) were conducted by female collaborators.

Before individual fishers were asked specific open-ended questions outlined in a questionnaire (see Supplementary Information S1 online), a casual conversation was initiated by the Fijian collaborator. Each interview was then started with an explanation about the main purpose of the survey. To facilitate and standardize the procedure, questionnaires included dichotomous and pre-categorized questions on target and bycatch shark fisheries, catch composition, fishing equipment, and use of sharks caught (see Supplementary Information S1 online). Fishers, both in the villages and at harbours in Suva and Lautoka, were asked to identify the species they catch or sell on the basis of an illustrative identification poster of the most common inshore elasmobranch species that occur in Fiji (http://fijisharkcount.com/the-activity/all-materials/id-posters/). We did not use and/or record traditional names for shark species and/or fishing gear. Anecdotal evidence was collected when possible regarding the trade in sharks and their products.

The study was conducted under a permit provided by the Fijian Ministry of Fisheries and Forests. All methods and protocols were approved by and carried out in accordance with instructions by the Principal Research Officer of the Department of Fisheries. Due to widespread illiteracy, a formal written consent was not possible for the majority of interviewees and therefore not pursued. Verbal consent was documented for all interviews in the respective questionnaire. All interviewed fishers collaborated on a voluntary basis, and data confidentiality and anonymity of each interviewee was assured.

## Tissue Sampling and DNA Barcode Protocol

Fishers who reported to target sharks and who had specimens with them at the time of the interview were asked whether a fin clip could be taken for species identification/verification. From this, a total of 21 tissue samples were collected from Momi (n = 2), Navakacoa (n = 4), Vitogo (n = 13), and Waya Levu (n = 2). In addition, seven muscle tissue samples were collected at local fish markets in Suva (n = 6) and Lautoka (n = 1) (Fig. 1a). In total 28 samples were collected for DNA barcoding from the catch of 11 interviewees. In each case, the interviewee was asked to identify the species based on morphology (Table 1).

Muscle and fin tissue were excised and preserved in 99% isopropanol. DNA was extracted following standard Phenol-Chloroform extraction (1x phenol, 1x phenol-chloroform-isoamylalcohol, 1x chloroform, adapted from Sambrook & Russel^51^), followed by precipitation of DNA with 0.1 volume 3M NaOAc and 3 volumes Ethanol^52^. Tissue samples were barcoded by sequencing the Cytochrome Oxidase I gene^53^. A 652-bp fragment from the 5’ region of the *cox1* gene from mitochondrial DNA was PCR amplified using FastStart Taq from Roche and FishF1 and FishR1^53^ (FishF1-5′TCAACCAACCACAAAGACATTGGCAC-3′, FishR1-5′TAGACTTCTGGGTGGCCAAAGAATCA-3′). PCR products were sequenced directly or cloned using TOPO TA Cloning Kit from Invitrogen and sequenced using BigDye Chemistry from Applied Biosystems on an ABI 3130xl sequencer (Applied Biosystems). Resulting sequences were identified by BLAST^54^ (http://blast.ncbi.nlm.nih.gov/Blast.cgi).

## Results

### Characterization of Artisanal Shark Fisheries in Fiji

A total of 253 interviews (11 females, 242 males) were conducted with interviewees between 18 and 80 years old (response rate = 100%). A total of 166 interviewees (65.6%) reported to have been fishing for between one (n = 1) and >60 years (n = 1), with slightly less than half (49.4%) for more than 20 years (see Supplementary Information S2 online).

**Figure 2:**
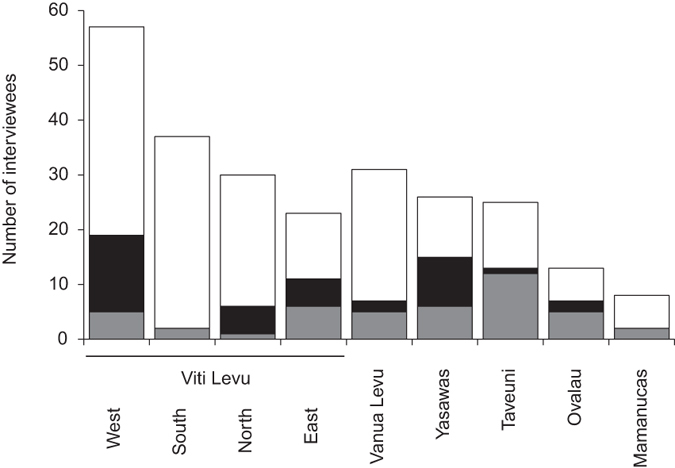
Geographic distribution of targeted and bycatch artisanal shark fisheries in Fiji. No sharks caught = grey bars; targeting sharks = black bars; sharks taken as bycatch = white bars.

Fishers reported catching sharks at all of the sites surveyed (Fig. 1a, c). Of the 253 interviewees, 206 (81.4%) reported to presently catch sharks, and 44 (17.4%) declared that they did not presently catch sharks (Fig. 1c, d). One interviewee said that he stopped catching sharks, and two said that they plan to catch sharks in the near future. Of the 206 fishers that reported to catch sharks, 38 (18.4%) targeted sharks, and 168 (81.6%) caught sharks as bycatch (Fig. 1e, f). While sharks were caught as bycatch throughout Fiji (Fig. 1f), targeted shark fisheries primarily occurred on Viti Levu with the exception of the south coast, and the Yasawa Islands (Figs. 1e and 2).

When asked about specific fishing grounds, of the 206 interviewees that reported to catch sharks (targeted and bycatch), 73.8% (n = 152) mentioned coral reefs, 45.6% (n = 94) the coastal zone, 38.4% (n = 79) beyond coral reefs, 4.4% (n = 9) rivers, 3.4% (n = 7) estuaries, and 0.5% (n =1) mangroves (note double entries are possible). When asked how many sharks they catch, the majority of fishers that catch sharks as bycatch reported to catch two or three sharks per week. In contrast, the majority of those that targeted sharks reported to catch three to six animals per person per week.

**Figure 3:**
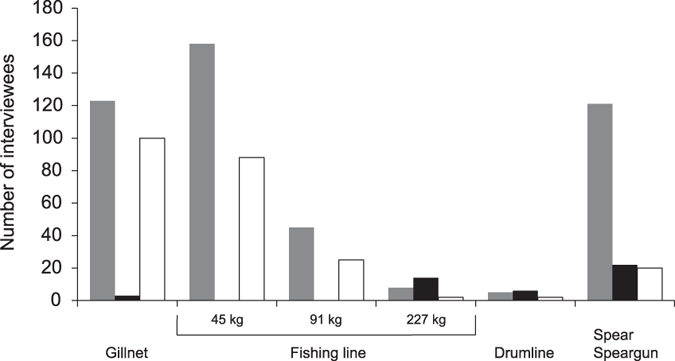
Fishing gears used to catch sharks. Gear used by Fijian fishers for fishing in general (grey bars), to target sharks (black bars) and when sharks are caught as bycatch (white bars).

While fishers reported to catch sharks with a variety of common fishing gears, gillnets and light to intermediate strength hand lines were the main gear types for the fishers that reported to catch sharks as bycatch (Fig. 3). In contrast, heavy lines, drumlines and handspears or spearguns were the main gear used to target sharks (Fig. 3). According to interviewees, spears and spearguns are ideal for catching mid-sized reef sharks as they are relatively abundant on coral reefs, whereas drumlines with catch capacities of around 150 to 250 kg are ideal for catching large sharks. Fishers stated that the use of a single drumline can lead to catches of up to 15–30 sharks per week.

**Figure 4:**
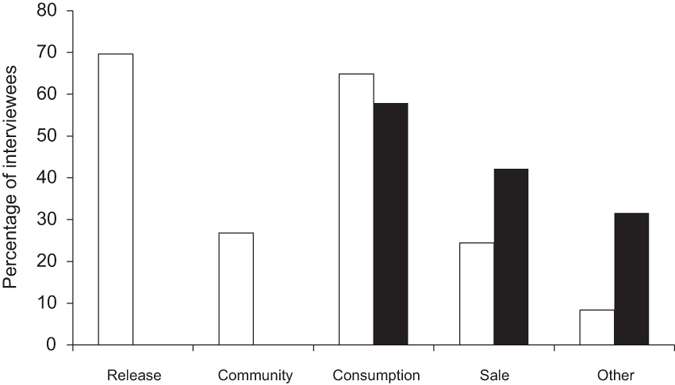
Use of sharks. Sharks caught in targeted fisheries (black bars) and taken as bycatch (white bars) are used for different purposes. Note double entries are possible.

**Figure 5:**
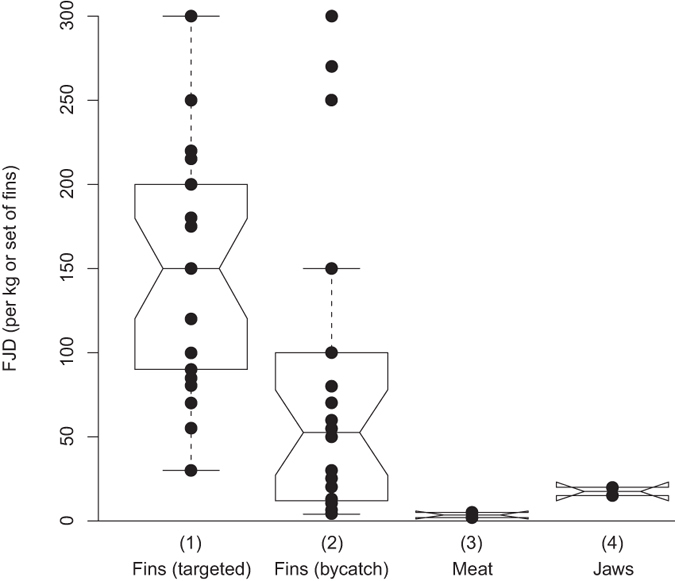
Revenues from sale of shark products. Box plots show the range of FJD earned per unit (kg or set of fins) from (1) the sale of shark fins by fishers targeting sharks (n = 34), (2) the sale of shark fins by fishers that take sharks as bycatch (n = 30), (3) the sale of shark meat (n = 4), and (4) the sale of jaws (n = 2).

Sharks caught as bycatch were reported to be utilised differently than those from targeted shark fisheries. Sharks that were targeted were used for local consumption and/or to sell (meat and fins; a few fishers mentioned that they also sell teeth and jaws) (Fig. 4). Six fishers (15.8%) reported to target/kill sharks to defend themselves and/or their catch. Sharks caught as bycatch were most frequently released dead or alive (69.6%), consumed (64.9%) or shared amongst the community (26.8%), and to a lesser extent sold (note double entries are possible) (Fig. 4). Fishers that reported to sell shark fins reported values of up to 300 FJD (161 USD) per kg of self-dried shark fins (Fig. 5). If sold, the revenues from sharks caught as bycatch were typically much lower than that from targeted shark fisheries (Fig. 5).

A total of 99 (46.7%) of the fishers that reported to either not catch sharks or catch them only as bycatch provided reasons for not targeting sharks. The majority were not interested in sharks for reasons such as sharks are taboo or because of religious beliefs (23.2%), they are prohibited from catching sharks, i.e. “authority regulations by government or village chiefs” (15.2%), they are Indo-Fijians (12.1%) or because they are interested in bony fishes and not sharks (12.1%) (Table 2).

### Species caught

Fijian fishers reported to catch at least 12 species of elasmobranchs, consisting of 11 sharks and one ray species (*Rhynchobatus australiae*). The main sharks caught were blacktip reef sharks *Carcharhinus melanopterus*, whitetip reef sharks *Triaenodon obesus*, various hammerhead shark species *Sphyrna* spp., bull sharks *Carcharhinus leucas* and tiger sharks *Galeocerdo cuvier* (Table 3). Whilst *T. obesus* and *C. melanopterus* were caught in all areas surveyed, *C. leucas* were caught predominantly around southern Viti Levu, and *Sphyrna* spp. predominantly in the south, west and north of Viti Levu (see Supplementary Information S3 online). Importantly, 45 (21.8%) of the interviewees reported to catch juvenile sharks of various species, primarily on Viti Levu (Table 3). Carcasses of juvenile sharks were frequently found at markets (see Supplementary Information S4 online).

During interviews and at fish markets, 28 tissue samples were collected from 11 interviewees for molecular identification and for verifying fishers’ identifications (Table 1). In 17 cases (60.7%), the interviewee identified the species correctly (Table 1). Sharks sampled in the fish market in Suva were landed by Chinese longliners and molecular barcoding results identified them as the shortfin mako *Isurus oxyrinchus*, oceanic whitetip shark *Carcharhinus longimanus*, scalloped hammerhead *Sphyrna lewini* and silky shark *Carcharhinus falciformis*; the mako being twice identified correctly, but the latter three species not identified correctly by the interviewee (Table 1). Although the two truly oceanic species, *C. longimanus* and *I. oxyrinchus*, are less likely to be encountered by artisanal fishers, they have been included in this study as they are entering the same market stream as the artisanal catches. One sample collected at the fish market in Lautoka was identified by the interviewee and confirmed with molecular identification as *S. lewini* (Table 1). *Carcharhinus albimarginatus* and *C. falciformis* were the most likely to be misidentified, while *T. obesus* was correctly identified in all cases (Table 1).

### Anecdotal Evidence Recorded During the Survey

Many fishers do not target sharks as they lack the knowledge on how to catch and process them, where to sell them, and generally what shark fins are used for. This pattern is supported by interviewees saying that fishers that catch sharks as bycatch often discard the whole animal at sea or the parts that are not edible (fins, head, guts) because they have no value for them. Some Fijian fishers stated that mostly Indian fishermen catch sharks to sell them at local markets.

Seven interviewees reported middlemen (mostly Chinese) coming to villages to encourage fishers to catch sharks and/or to collect shark fins. Although two of the interviewees reported that their interest in shark fins was relatively consistent in recent years, four interviewees noted that interest in shark fins increased in recent years. Three interviewees in villages on Viti Levu, Vanua Levu and Taveuni said that middlemen were primarily interested in *bêche-de-mer* (dried sea cucumbers), but switched to shark fins if *bêche-de-mer* was not available. The main hubs for the shark (fin) trade in Fiji were reported to be Lautoka (local traders) and Suva (high sea fleets).

Anecdotal reports also suggested that seafood companies offer fishing and SCUBA diving gear to fishers to catch sharks in some instances. Interviewees also reported to receive hooks and lines from relatives that work on commercial fishing vessels. However, we also collected anecdotal reports that there was increasing awareness regarding sharks and their role in coastal ecosystems due to awareness programmes run by authorities and non-governmental organizations.

## Discussion

This study presents the first focused exploration of the shark catch in Fiji’s artisanal coastal fisheries and provides information on fishing gear used and motivations to catch or release sharks. The interviews were a rich source of information confirming that various reef shark species are regularly caught in inshore fisheries. The underlying motivations for shark fishing were found to be dynamic. Whereas some fishers target sharks, the majority take them as bycatch. Sharks are mainly used for personal consumption, but there is some anecdotal evidence which suggests that the international shark fin trade is possibly exerting an increasing amount of pressure on local reef shark stocks.

Sharks are not considered an important source of food in many parts of Fiji, mainly due to traditional food taboos which possibly serve to protect against dangerous marine toxins^39^,^55^. However, the meat from several species is known to be eaten in areas where it is not taboo^56^. Our findings show that domestic consumption is the main use of sharks caught both in targeted and bycatch shark fisheries. Taking sharks for personal consumption and/or selling them at local markets may be the result of local demand for alternative food fishes due to the loss of traditional fish stocks^39^. Hence, Fijian shark fisheries can be regarded as subsistence in nature, i.e. sharks are harvested for domestic consumption.

Anecdotal evidence was found in this study that Fijian artisanal fishers only recently began targeting sharks to sell their fins to Chinese middlemen who visit the villages or directly to seafood companies situated in and around Lautoka and Suva. This is similar to *bêche-de-mer* fisheries where *bêche-de-mer* collected at localities close to urban centres are more frequently directly sold to middlemen or to export companies^7^. Results from the interviews conducted in this study showed that the *bêche-de-mer* and shark fin trades are linked, i.e. the demand for shark fins was reported to increase when less dried sea cucumbers are available. Asian traders looking for new species and/or fishing grounds have been recorded as having instigated new fisheries^7^. Hence, it is reasonable to assume that the artisanal shark fisheries in Fiji are using the well-established trade chains of the *bêche-de-mer* fisheries^7^.

The shark species most commonly caught were *C. melanopterus* and *T. obesus*, both of them listed as Near Threatened on the IUCN Red List^57^. *Carcharhinus melanopterus* and *T. obesus* show pronounced long-term site fidelity to specific small-scale coastal or reef habitats^58^,^59^,^60^,^61^. Hence, even low fishing pressure may remove these apex predators permanently from particular reefs which may result in cascading effects^62^ since typically the largest reef fish, i.e. sharks, are especially vulnerable^63^.

The only ray species reported to be caught, and confirmed by DNA barcoding, in this study was *R. australiae*. This species has been assessed by *The IUCN Red List of Threatened Species* as Vulnerable^57^. *Rhynchobatus* spp. are considered to be among the most vulnerable elasmobranchs (sharks, skates and rays) due to their life-history characteristics and high-valued fins^64^,^65^. Prior to this study, the presence of this genus from Fiji was only based on anecdotal accounts, so the sample of *R. australiae* collected in Momi on the western coast of Viti Levu is the first confirmed record of this species in Fiji’s inshore waters (see Supplementary Information S5 online).

Every person we approached agreed to be interviewed. This is a strong indication that we had acceptance and trust of the community, hence increasing our confidence in the survey responses. Nevertheless, one limitation of our study is that correct species identification by local fishers is an ongoing issue in most developing countries. Whereas using an illustrative identification poster may be helpful for fishers to identify the species correctly, not all the species that occur in the respective area may be featured on the poster. This highlights the need for regional specific guides which provide images of species and list the key features which separate similar species. Our study acts as a baseline from which such regional guides can and should be produced to alleviate the problems with correct shark species identification in Fiji. Although we cannot be sure that all interviewees identified species correctly in all cases (Table 3), by verifying a number of identifications using DNA barcoding, we were able to determine that the majority of fishers identified species correctly, in particular the most common ones (Tables 1 and 3). Hence, we are confident that we have captured a reliable picture of what shark species are caught most commonly in Fiji’s artisanal shark fisheries.

In Fiji, marine resources are traditionally controlled by local social units, such as clans and villages^66^. The country recently decided against a National Shark Sanctuary which would have prohibited commercial shark fishing, import, export and sale of shark products^67^. Hence, no management measures for sharks exist at the national level and artisanal shark fisheries remain to be managed at the community level on the basis of a well-established marine tenure system^68^,^69^,^70^. There is evidence that this system has been destabilized due to the commercialization of fishing activities, for example, by turning previously restricted coastal resources into open-access fisheries^71^.

Our study provides data on the extent of Fiji’s artisanal shark fisheries. In general, they are small but could possibly develop into larger and potentially more targeted fisheries. This is supported by our finding that fins from targeted shark fisheries reportedly fetch higher prices compared to fins sold from sharks caught as bycatch (Fig. 5). Higher revenues from targeted sharks may motivate fishers to catch specifically larger sharks with more valuable fins using the respective gear. In order to obtain more data to allow informed fishery management decisions, Fiji’s fisheries authorities should consider establishing monitoring programmes for sharks. Such programmes would provide much-needed information on changes in shark abundance, and catch and size composition through active monitoring, e.g. market surveys. These programmes would also need to include socioeconomic assessments of the fishery in order to identify social drivers that may lead to the unsustainable use of local shark stocks. Changes in trade of various fish products, such as shark fin, can be the main driver for changes in artisanal fisheries. For example, a buyer with good export linkages can offer more money for dried shark fins and thus drive fishers to increase targeting of sharks. These data can be used to assess the impacts and risks of these fisheries and to determine their level of sustainability. The fact that juvenile *S. lewini* are often sold in local fish markets (see Supplementary Information S4 online) confirms that, for at least some species, all life stages from neonatal to adult sharks are affected by artisanal shark fisheries.

Two species observed for sale in fish markets in Fiji (*S. lewini* and *C. longimanus*) are listed on Appendix II of CITES as they are at the risk of becoming threatened with extinction unless trade is closely controlled. Another species (*I. oxyrinchus*) is listed on Appendix II of the Convention on the Conservation of Migratory Species of Wild Animals (CMS) which Fiji has been a party to since 2013. Careful monitoring of these species is necessary to ensure that the way these species are exploited and traded complies with the relevant CITES and CMS obligations and should be considered as a driver to improving national and regional management of these important apex predators.

Lastly, future studies on Fiji’s inshore shark fisheries should also include interviews with traders, exporters or market sellers. These other sources higher up the fisheries value chain could provide valuable data on the shark fishery and trade. Such data could also help to validate the main findings of our study about trade and fishery trends. Understanding the social, cultural and economic drivers that are the reasons people fish for, sell and trade in sharks in the way they do is vital in planning conservation and management.

## Additional Information

**How to cite this article**: Glaus, K. B. J. *et al.* Characteristics of the shark fisheries of Fiji. *Sci. Rep.* **5**, 17556; doi: 10.1038/srep17556 (2015).

## Supplementary Material

Supplementary Information

## Figures and Tables

**Table 1:** Comparison of elasmobranch species identified using DNA barcoding (COI gene), and by interviewees.

Interviewee	Origin	Species (barcoding)	Species (interview)
Trader 1	Suva (fish market)	*Isurus oxyrinchus*	Mako
Trader 2	Suva (fish market)	*Isurus oxyrinchus*	Blue shark
Trader 3	Suva (fish market)	*Isurus oxyrinchus*	Mako
Trader 4	Suva (fish market)	*Carcharhinus falciformis*	“brown shark”
Trader 5	Suva (fish market)	*Sphyrna lewini*	“brown shark”
Trader 6	Suva (fish market)	*Carcharhinus longimanus*	Mako
Trader 7	Lautoka (fish market)	*Sphyrna lewini*	Hammerhead
68	Momi	*Rhynchobatus australiae*	Guitarfish
68	Momi	*Nebrius ferrugineus*	Nurse shark
189	Waya Levu	*Galeocerdo cuvier*	Bull shark or Tiger shark
189	Waya Levu	*Galeocerdo cuvier*	Bull shark or Tiger shark
121	Navakacoa	*Carcharhinus amblyrhynchos*	Grey shark
121	Navakacoa	*Carcharhinus albimarginatus*	Whitetip shark
121	Navakacoa	*Carcharhinus albimarginatus*	Whitetip shark
121	Navakacoa	*Carcharhinus albimarginatus*	Mako
179	Vitogo	*Carcharhinus melanopterus*	Blacktip shark
179	Vitogo	*Carcharhinus amblyrhynchos*	Bull shark
179	Vitogo	*Sphyrna lewini*	Hammerhead
179	Vitogo	*Carcharhinus melanopterus*	Blacktip shark
179	Vitogo	*Carcharhinus falciformis*	Tiger shark
179	Vitogo	*Carcharhinus falciformis*	Tiger shark
179	Vitogo	*Carcharhinus falciformis*	Tiger shark
179	Vitogo	*Nebrius ferrugineus*	Bull shark
179	Vitogo	*Nebrius ferrugineus*	Bull shark
179	Vitogo	*Triaenodon obesus*	Whitetip shark
179	Vitogo	*Triaenodon obesus*	Whitetip shark
179	Vitogo	*Triaenodon obesus*	Whitetip shark
179	Vitogo	*Triaenodon obesus*	Whitetip shark

Blast coverages of the query sequences ranged from 97% to 100%, percent identity with the identified sequences ranged from 98% to 100%. Identification using the BOLD-database was unambiguous in all cases.

**Table 2:** Reasons (number (#) and percentage (%) of interviewees) fishers (n = 99) gave for not targeting sharks.

**Reason**	**#**	**%**
Taboo/totem/religion	23	23.2
*Shark is a family totem*; *It is taboo to catch them due to ancient beliefs/religion*; *Sharks are taboo to eat because consumption of their meat might lead to skin disease; the skin gets white stains and finally turns into white*.		
Prohibited	15	15.2
*Prohibited by law*; *Prohibited by village chief because he is aware of the environmental function of sharks due to awareness programmes or because sharks are a totem of the village chief’s family*.		
Ethnos	12	12.1
*I am Indo-Fijian and Indian fishermen do not eat shark meat*.		
Interested in fish not sharks	12	12.1
*It never came to mind to catch and eat sharks when other fishes are around*.		
Opportunity	10	10.1
*I do not come across sharks*; *I don’t know what fishing gear to use to catch sharks*; *I don’t have my own fishing boat that would allow me to catch sharks*.		
Awareness	9	9.1
*Taught by NGOs about the environmental function of sharks*.		
Taste	6	6.1
*I don’t like the taste of shark meat*; *Shark meat is low quality*.		
Habit/tradition	5	5.1
*I’m not used to catch sharks*; *I use to only catch bony fishes*.		
No access	4	4
*I don’t have access to areas where sharks can be found*.		
Fear of sharks	3	3
*It is difficult and dangerous to catch sharks*.		

Examples of typical answers are given in italics below the reason.

**Table 3:** Number (#) and percentage (%) of interviewees (total = 206) that reported to catch the respective species.

Species	#	%
*Carcharhinus melanopterus*	112	54.4
*Triaenodon obesus*	100	48.5
*Sphyrna* spp.	78	37.9
*Carcharhinus leucas*	49	23.8
*Galeocerdo cuvier*	39	18.9
*Rhynchobatus australiae*	16	7.8
*Nebrius ferrugineus*	15	7.3
*Carcharhinus amblyrhynchos*	13	6.3
*Stegostoma fasciatum*	9	4.4
*Negaprion acutidens*	5	2.4
*Isurus oxyrinchus*	3	1.5
*Carcharhinus albimarginatus*	3	1.5
unknown	34	16.5
any species	15	7.3
juveniles (various species)	45	21.8
