# Supplementary material for: Characteristics of the shark fisheries of Fiji
Source: Sci Rep. 2015 Dec 2;5:17556. doi: 10.1038/srep17556 (PMC4667191; doi:10.1038/srep17556)

Supplementary Information

for

Characteristics of the shark fisheries of Fiji

Kerstin B. J. Glaus, Irene Kalchhauser, Patricia Burkhardt-Holm,
William T. White & Juerg M. Brunnschweiler*

* Corresponding author
E-mail: juerg@gluecklich.net

Supplementary Information S1

Questionnaire used during the interviews

Artisanal Shark Fishing
For how many years have you been a fisher?
What do you usually catch? (only fish, crabs, prawns, bêche-de-mer)
Where do you usually go fishing? (rivers, esturians, coastel zone, pelagic zone, coral reefs)
Do you fish specifically for sharks?
If yes, how many sharks do you catch per time unit? (day, week, month)
What types of sharks do you usually catch?
Where do you fish for sharks? (rivers, estuaries, coastal zone, pelagic zone, coral reefs)?
Do you fish for sharks seasonally?
What shark species do you catch?
Do you fish for rays?

Purpose of shark catching
For what purpose do you catch sharks? (self-consumption, sale, export, artifacts, handicrafts)
Do you consume sharks? If yes, how often?
If no, why not?
Which species do you usually consume?
Which parts of them do you consume?
Do you sell sharks / shark products?
If yes, which products do you sell?
What is the price for the sharks / shark products?
Who is interested in buying sharks / shark products from you?
For which purpose do they buy the sharks / shark products?
Are more people interested in buying sharks / shark products nowadays than earlier?
Have you ever been approached by people to catch sharks for them?
Do you know any fishermen who catch sharks specifically?

Bycatch
Do you have sharks as bycatch?
If yes, are they dead or alive when you find them?
How often and which shark species do you have as bycatch?
If you have sharks as bycatch, what are you doing with them? (self-consumption, selling)
If you sell them, who is interested in buying?
Which shark products are they buying and for which purpose?

Fishing Techniques
What fishing gear / technique do you usually use?
What is the best method to catch sharks? Why?
How many boats in total exist in your village?

Fish Traders
Which shark species do you usually sell?
Where were these sharks caught?
How where these sharks caught?
Who caught these sharks?
When were these sharks caught?
How much do you pay for one portion of shark meat?
Who are the buyers of the shark meat you are offering?
How much sharks/portions do you sell per day/week and what do they cost?

Supplementary Information S2

A total of 166 interviewees (157 males, 9 females) reported to have been fishing for between one and >60 years. The numbers in/above the bars indicate the number of female fishers in the respective category.

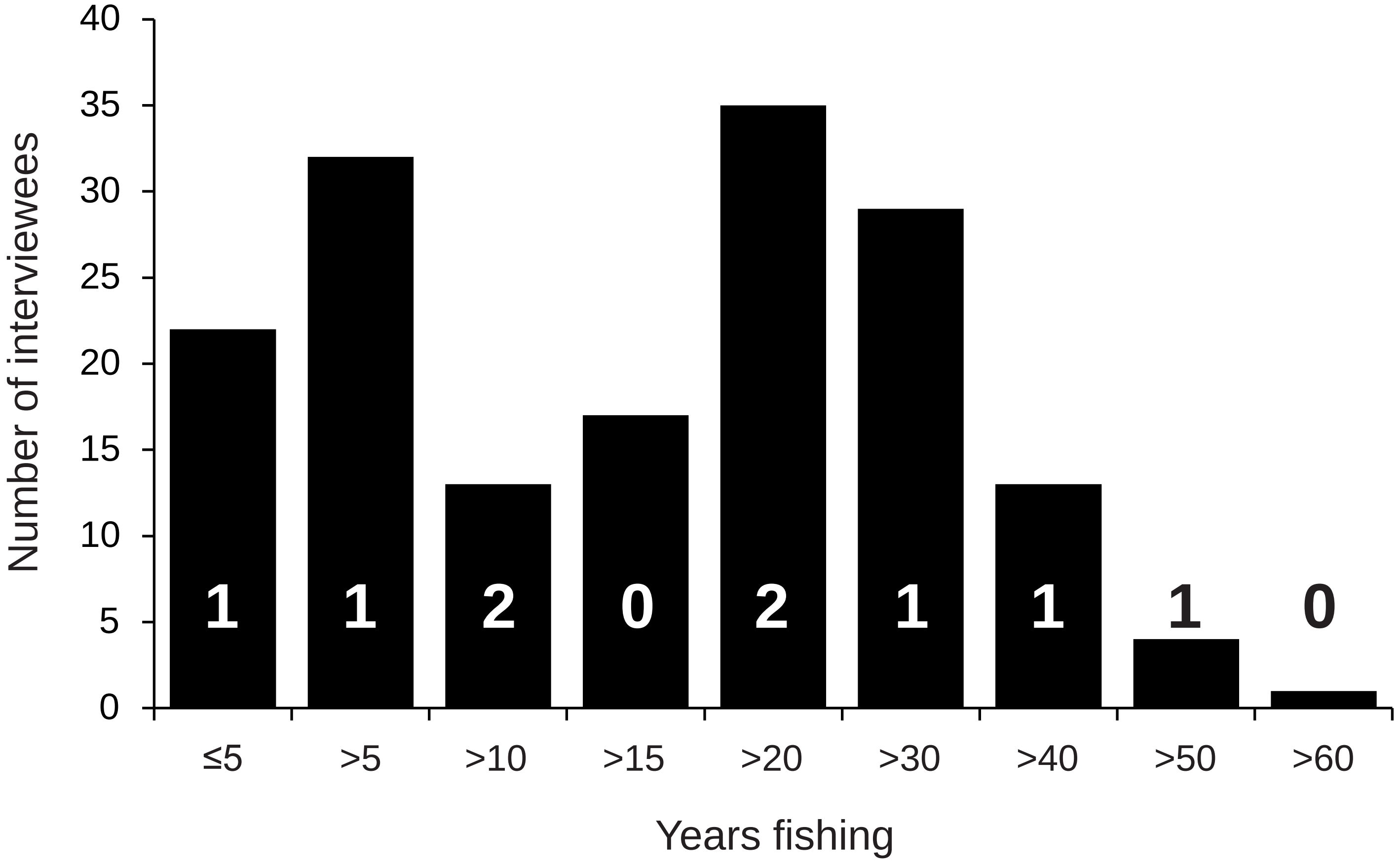

Supplementary Information S3

Bubble plots of the number of interviewees that reported catching the respective species (targeted and bycatch). The area of each dot corresponds to the number of interviewees at this site (see first panel *C. melanopterus* for scale of bubble sizes). Maps were generated using the map function of R on publicly available coastline coordinates obtained from the NOAA National Geophysical Data Center (http://www.ngdc.noaa.gov/mgg/shorelines/shorelines.html).

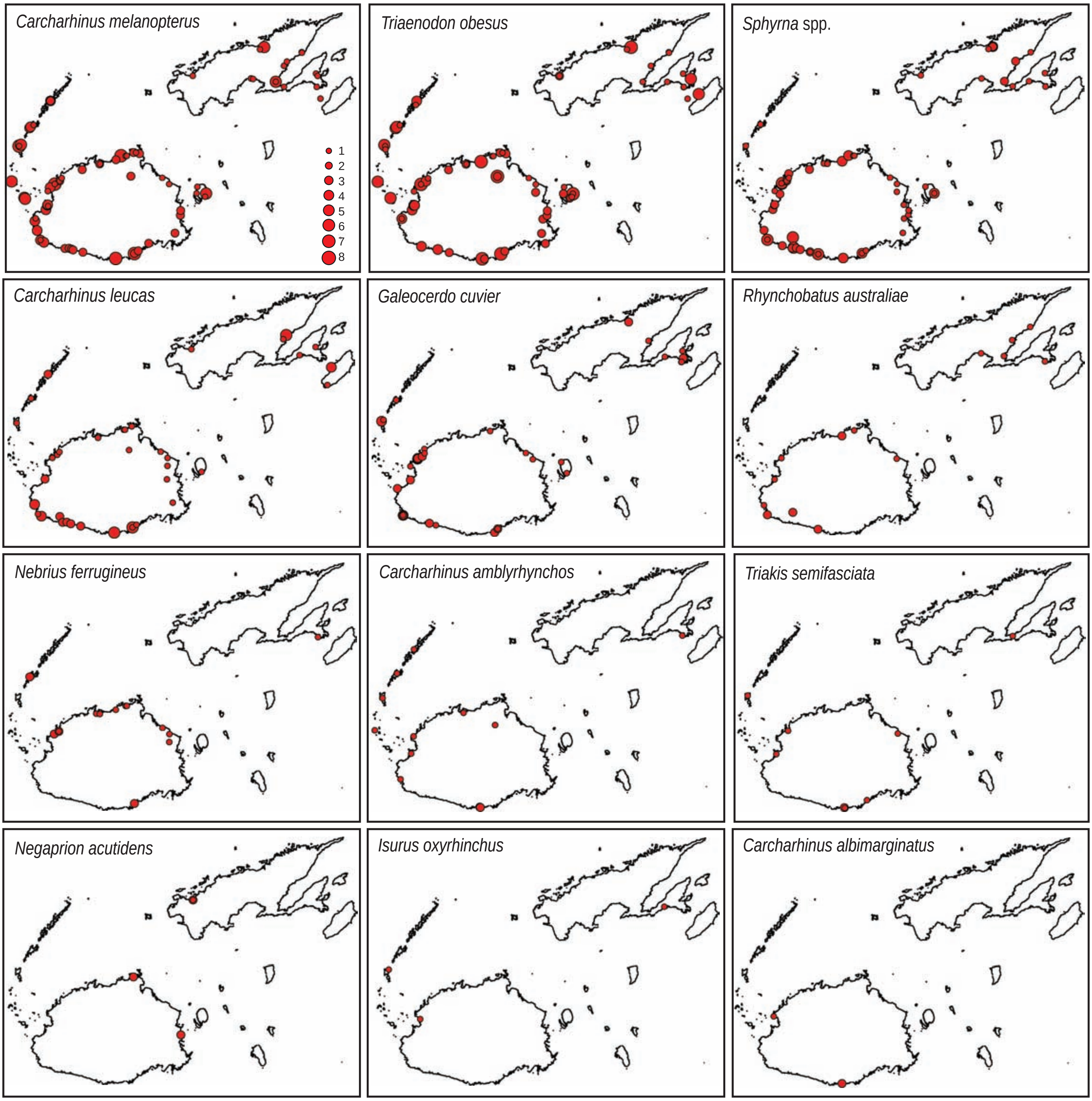

Supplementary Information S4

Juvenile *Sphyrna lewini* at the fish market in Lautoka. Photographs taken in February 2013 by Kerstin Glaus.

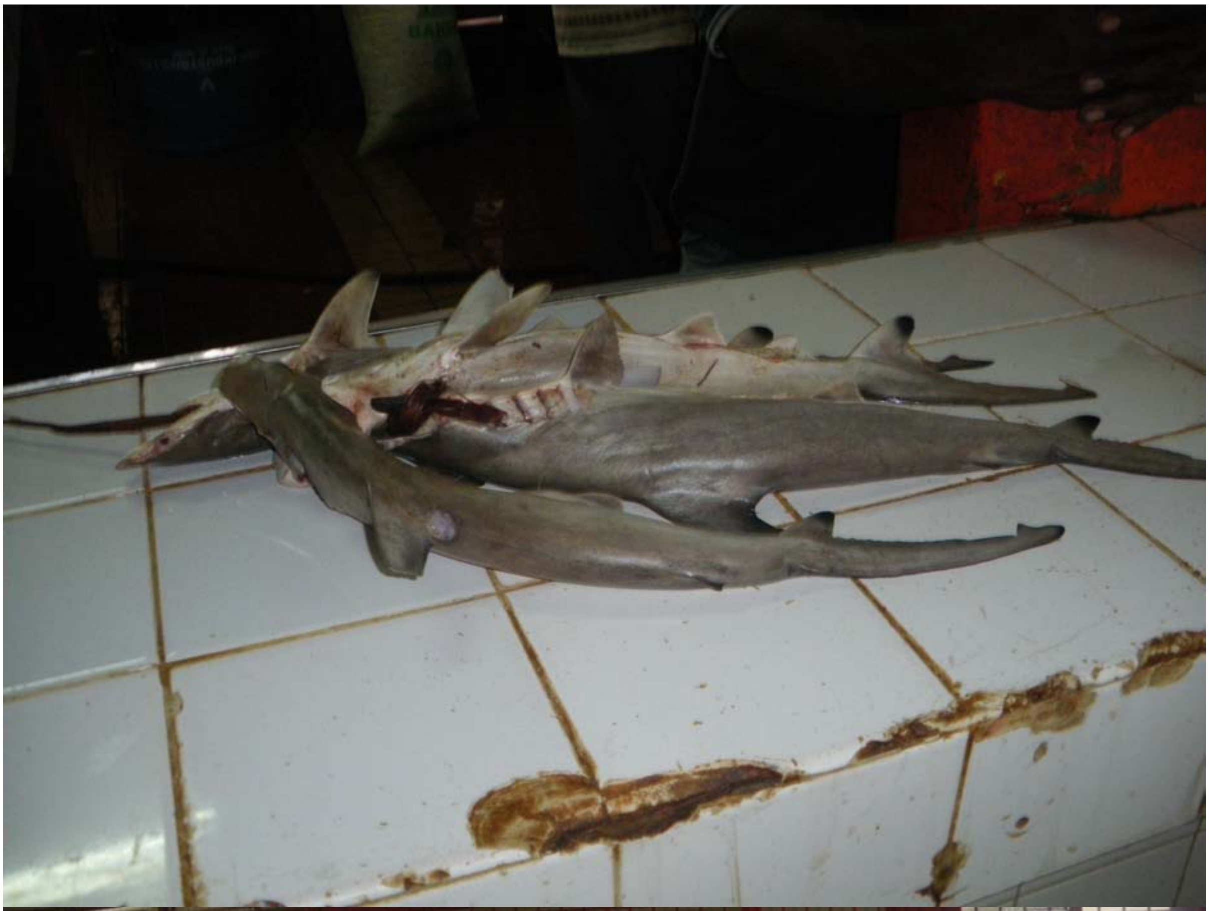

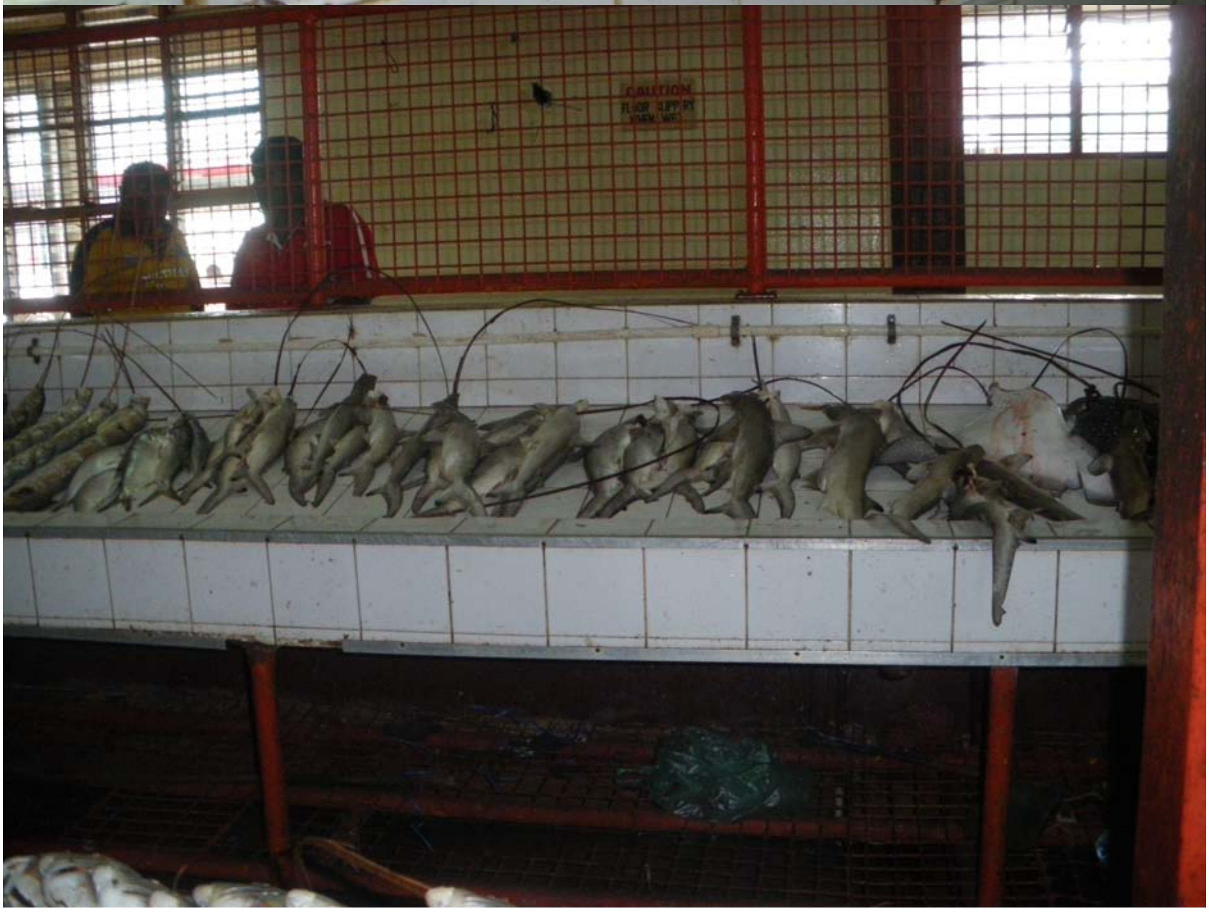

Supplementary Information S5

Dorsal and lateral view of *Rhynchobatus australiae* from Fiji. Photographs taken in January 2008 by Juerg Brunnschweiler.

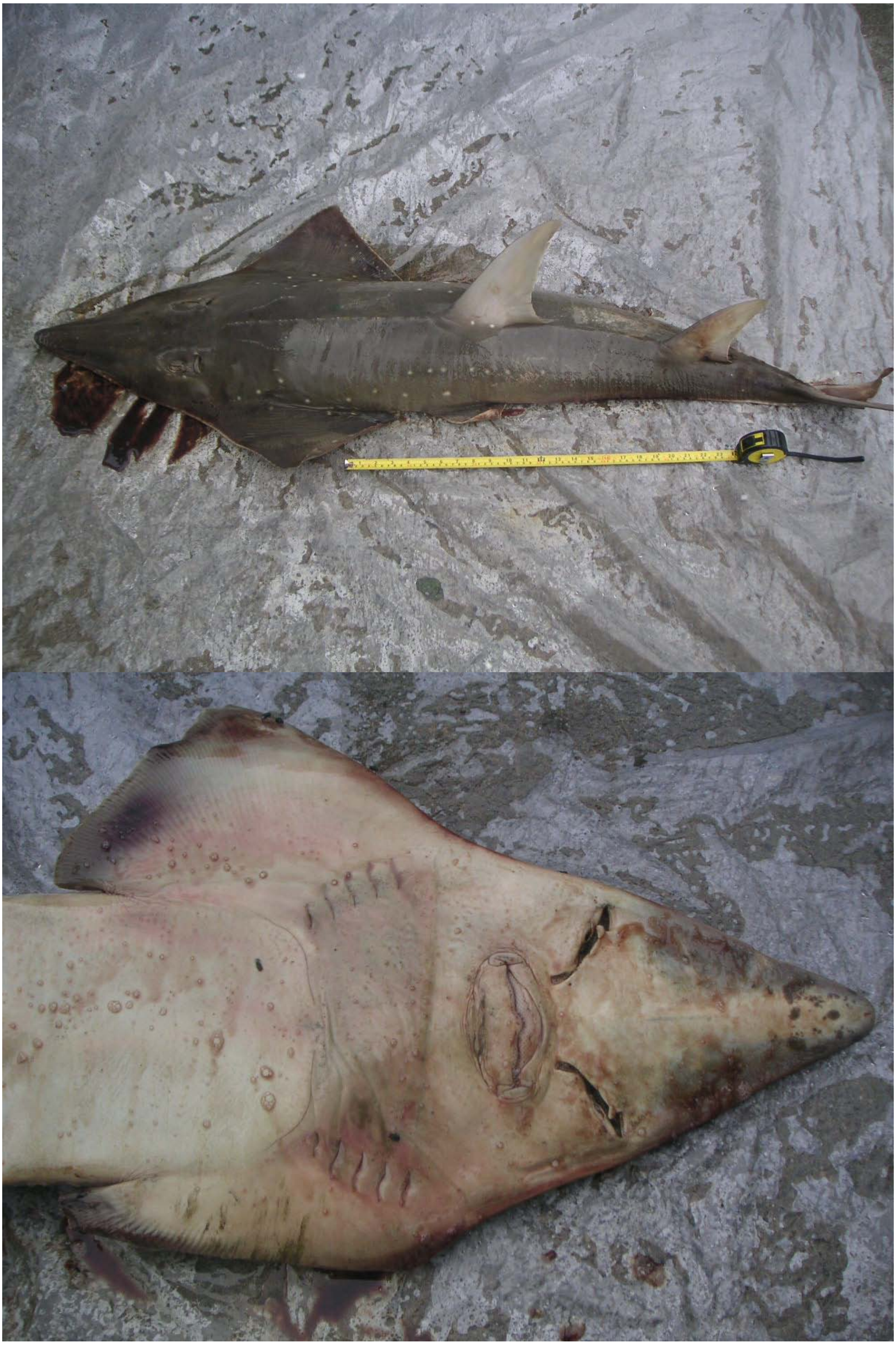

Supplement: Supplementary Information [file srep17556-s1.pdf]
